# Cost as a Prohibitive Factor on Effectiveness of Informational Campaigns to Reduce Dental Sealant Disparities

**Published:** 2005-06-15

**Authors:** Kenneth Anthony Bolin

**Affiliations:** Department of Public Health Sciences, Baylor College of Dentistry, Dallas, Tex

To the Editor:

The authors of the abstract "Reducing Dental Sealant Disparities in School-aged Children Through Better Targeting of Informational Campaigns" (1) state:

The lack of an association between sealant prevalence and knowledge among low-income families may reflect higher levels of public provision of sealants to this group. This suggests that informational campaigns could increase demand for sealants in both income groups.

However, I fail to see how targeted informational campaigns about sealants, regardless of the efficiency or the impact, could increase demand for a service in groups that cannot afford that service.
